# Supplementary material for: Macroscopic Poly Schiff Base-Coated Bacteria Cellulose with High Adsorption Performance
Source: Polymers (Basel). 2020 Mar 23;12(3):714. doi: 10.3390/polym12030714 (PMC7183273; doi:10.3390/polym12030714)
Supplement: Supplementary file 1 [file polymers-12-00714-s001.zip › polymers-750601-supplementary.docx]

Supplementary material

Macroscopic poly Schiff base-coated bacteria cellulose with high adsorption performance

Lili Ren ^a^, Zhihui Yang ^a,b^, Lei Huang ^a^, Yingjie He ^a^, Haiying Wang* ^a,b^, Liyuan Zhang* ^c,d^

^a^ School of Metallurgy and Environment, Central South University, 410083 Changsha, Hunan, PR China

^b^ Chinese National Engineering Research Center for Control and Treatment of Heavy Metal Pollution, Central South University, Changsha 410083, China

^c^ Department of Colloid Chemistry, Max Planck Institute of Colloids and Interfaces, 14476 Potsdam-Golm, Germany

^d^ Environmental Engineering Research Centre, Department of Civil Engineering, The University of Hong Kong, Pokfulam, Hong Kong

Correspondence: [haiyw25@163.com](mailto:haiyw25@163.com), [lyzhang@mpikg.mpg.de](mailto:lyzhang@mpikg.mpg.de), [zhang_livyl@csu.edu.cn](mailto:zhang_livyl@csu.edu.cn).

Corresponding Author: *H.W. and *L.Z.

Content

[1. Detail of Fabrication 3](#_Toc35221383)

[1.1 Pretreatment of BC 3](#_Toc35221384)

[1.2 Fabrication of BC/Schiff base composites 3](#_Toc35221385)

[1.3 The method to determine the concentration of pollutant 3](#_Toc35221386)

[2. Photograph of pBC-Polym-0.04 and BC-Polym-0.04 4](#_Toc35221387)

[3. The morphology of pBC-Polym-0.02 and pBC-Polym-0.08 4](#_Toc35221388)

[4. Specific surface area and porosity 5](#_Toc35221389)

[5. Thermal stability 6](#_Toc35221390)

[6. Effect of pH 7](#_Toc35221391)

[7. Adsorption mechanism 8](#_Toc35221392)

[8. Other pollutants 9](#_Toc35221393)

[Reference 9](#_Toc35221394)

1. Detail of Fabrication

1.1 Pretreatment of BC

The pristine bacteria cellulose (BC) pellicles was biosynthesized via static cultivation of bacterial in HS medium (consisted of 20g D-glucose, 5g yeast extract, 5g peptone, 1.15g citric acid monohydrate, 2.7g disodium hydrogen phosphate per 1 L and autoclaved at 115°C for 20 min prior inoculation) at 26 ºC for 7 d. The BC pellicles harvested at interface of air and liquid was cut down into the cubic block (1cm length). Then the blocky BC was treated with the mixture of 1 M sodium silicate solution (Na_2_SiO_3_·12H_2_O) solution and 1 M sodium carbonate (Na_2_CO_3_) solution (V: V= 1: 1) at 70 ºC to allow the silicate and carbonate diffusing into BC matrix and concurrently purging the residual cell and medium. Afterward, accompanied by gas of carbon dioxide releasing, the silica was deposited in BC hydrogel by immersion in 2 M hydrochloride acid (HCl) under ultrasonication for 12 h. After reaction, the BC-silica composite sank in 2 M sodium hydroxide (NaOH) solution to remove the silica thoroughly. Via rinsing by deionized water to neutrality and solvent-exchange with *turt*-butanol, the pretreated BC (short for pBC) was achieved by freeze-drying at -65 ºC for 24 h. Another BC block sample was treated with s NaOH before washed by deionized water to neutrality and freeze-dried as same approach, named as BC.

1.2 Fabrication of BC/Schiff base composites

A pH-controlled Schiff base reaction was applied in our experiment to prepare the precursor solution. Briefly, a certain amount of m-phenylenediamine (mPD) monomer was dissolved in 200 mL deionized water with adding double molar amount of hydrochloric acid by droplet. Following pouring equal molar amount of glutaraldehyde (GA) with mPD by magnetically stirred in ice bath, 1g pBC sample was immediately soaked in the mixture for 4 h under 4 ºC to absorb the soluble oligomers generated by GA and protonated mPD. Afterwards, the coated pBC was immersed in the 2 M NaOH for 24 h to synthesize the pBC/poly Schiff base composites and then rinsed repeatedly by water until the flushing fluid was neutral. The reduction of composites was proceeded in 200 mL 0.4 mol L-1 Sodium borohydride (NaBH_4_) solution under 80 ºC for 8 h. Finally, the reductive pBC/poly Schiff base aerogels were gained after removing the redundant reductant by ultrasonically washing in water and freeze-drying at -65 ºC for 24 h. For the sake of contrast, the BC/poly Schiff base aerogels were prepared by the same procedure using the BC aerogel as the feedstock.

1.3 The method to determine the concentration of pollutant

The Cr(VI) concentration of filtrate was determined by a UV-visible spectrophotometer (Hitachi U-4100) at 540 nm using 1,5-diphenylcarbohydrazide spectrophotometric method while the concentration of total Cr was measured by an inductively coupled plasma (ICP) emission spectroscopy (ICP-OES, Agilent 5100 SVDV, Santa Clara , CA, USA ).

The concentration of Re(VII) and Cu(II) solution was determined by an inductively coupled plasma (ICP) emission spectroscopy while the concentration of Conga red and Orange G was measured utilizing a spectrophotometric method at the wavelength of 498 nm and 478 nm, respectively.

2. Photograph of pBC-Polym-0.04 and BC-Polym-0.04


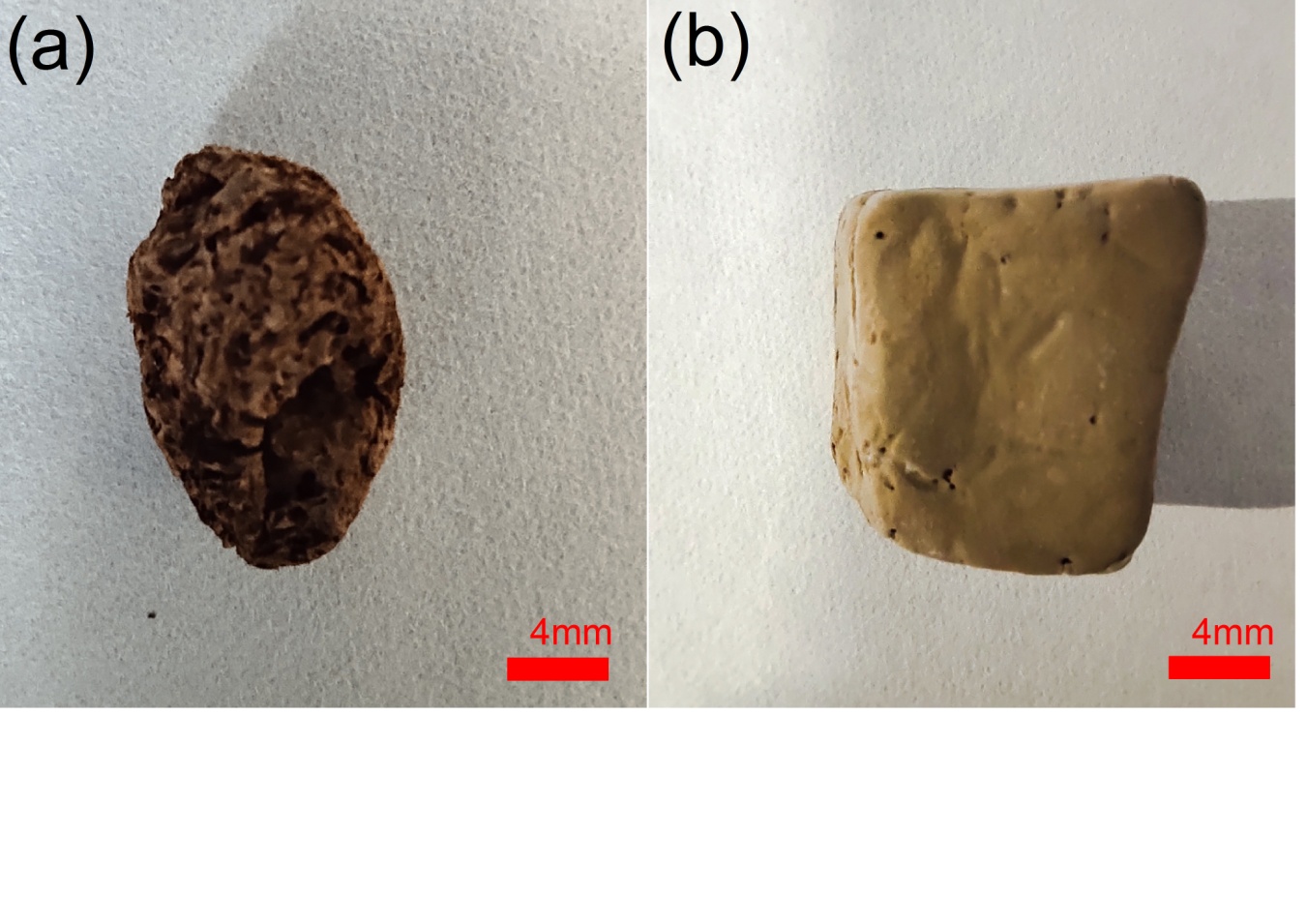


**Figure S1.** The photograph of pBC-Polym-0.04 **(a)** and BC-Polym-0.04 (**b**).

3. The morphology of pBC-Polym-0.02 and pBC-Polym-0.08


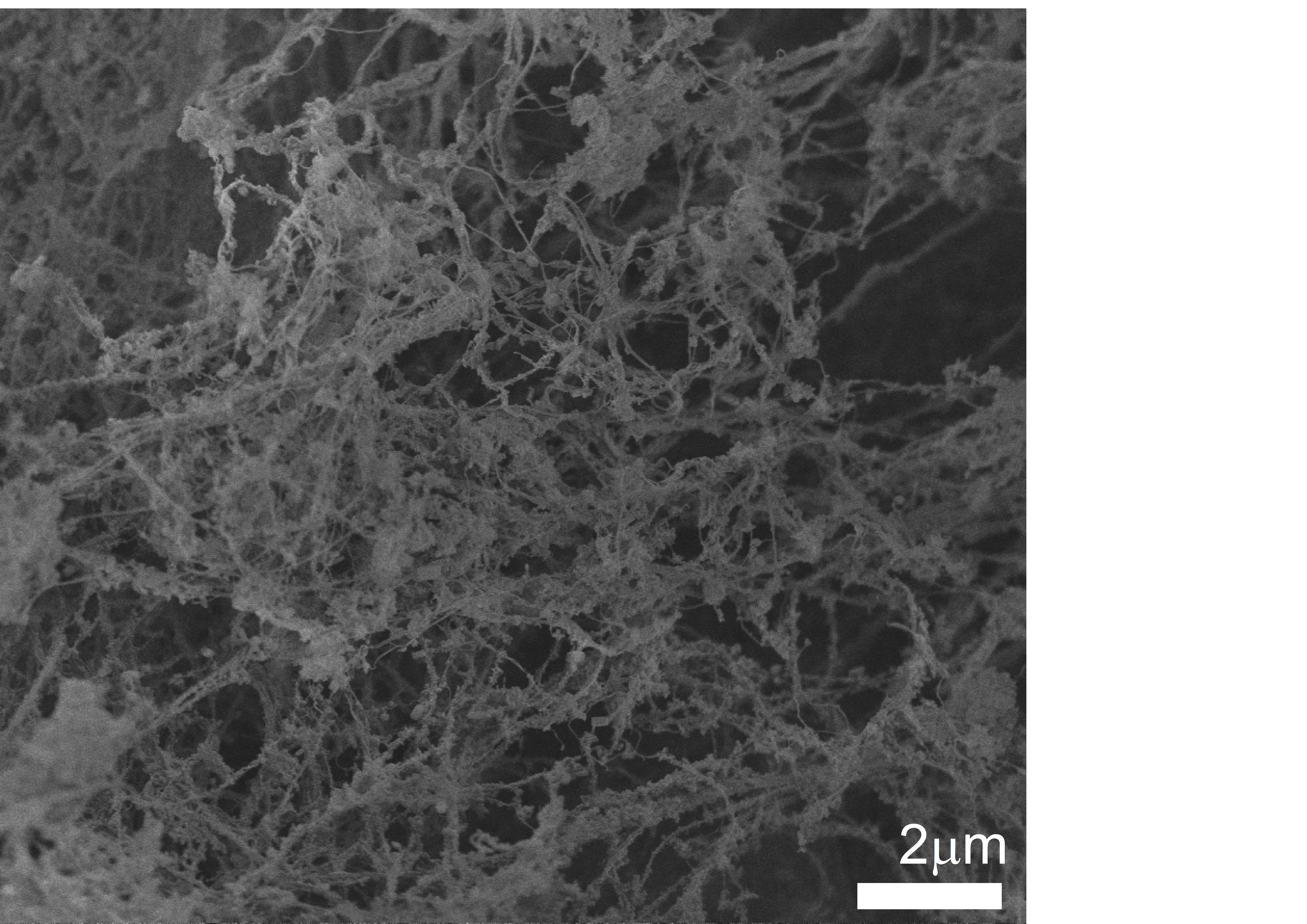


**Figure S2.** The SEM image of pBC-Polym-0.02.

**
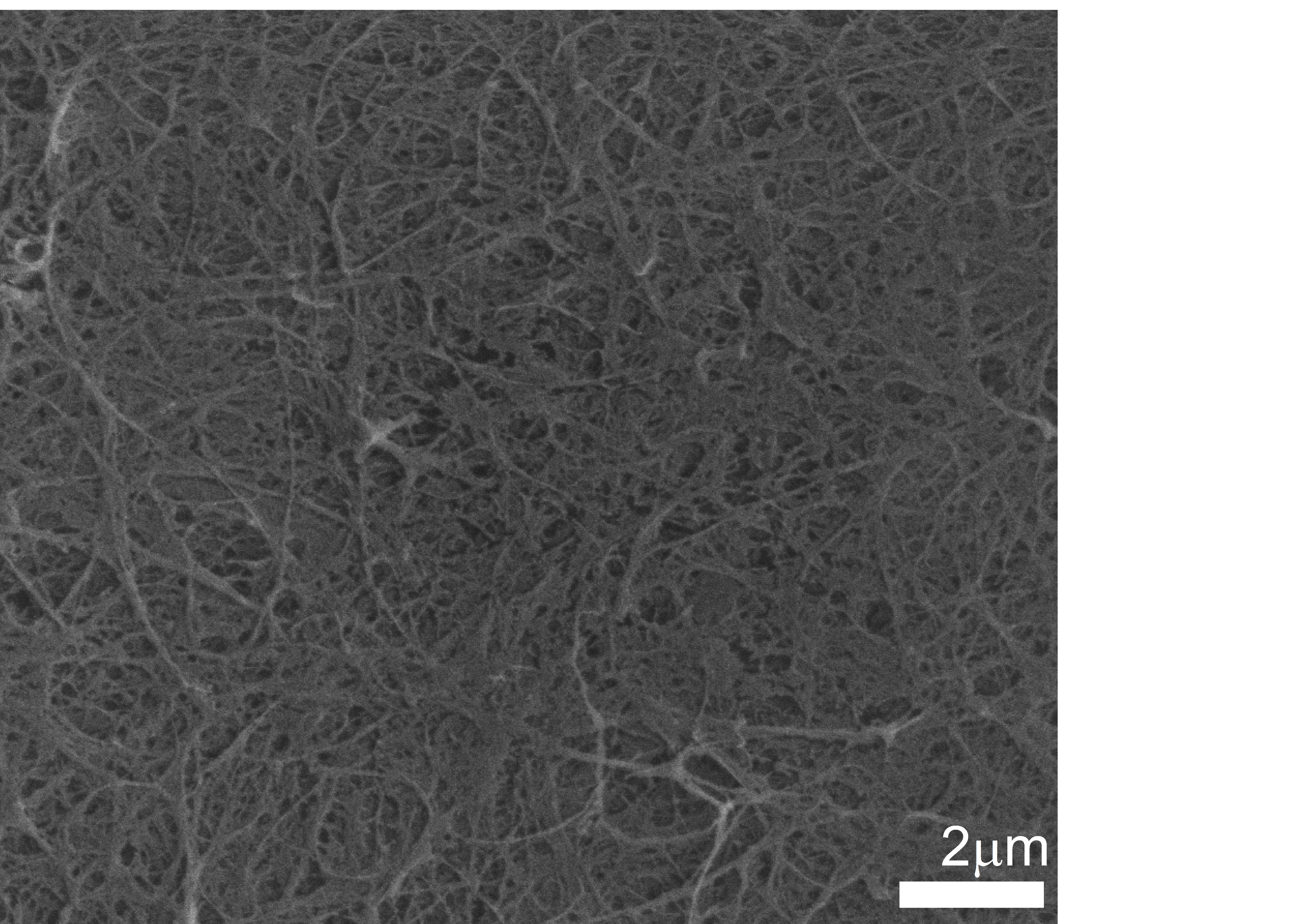
**

**Figure S3.** The SEM image of and pBC-Polym-0.08.

4. Specific surface area and porosity


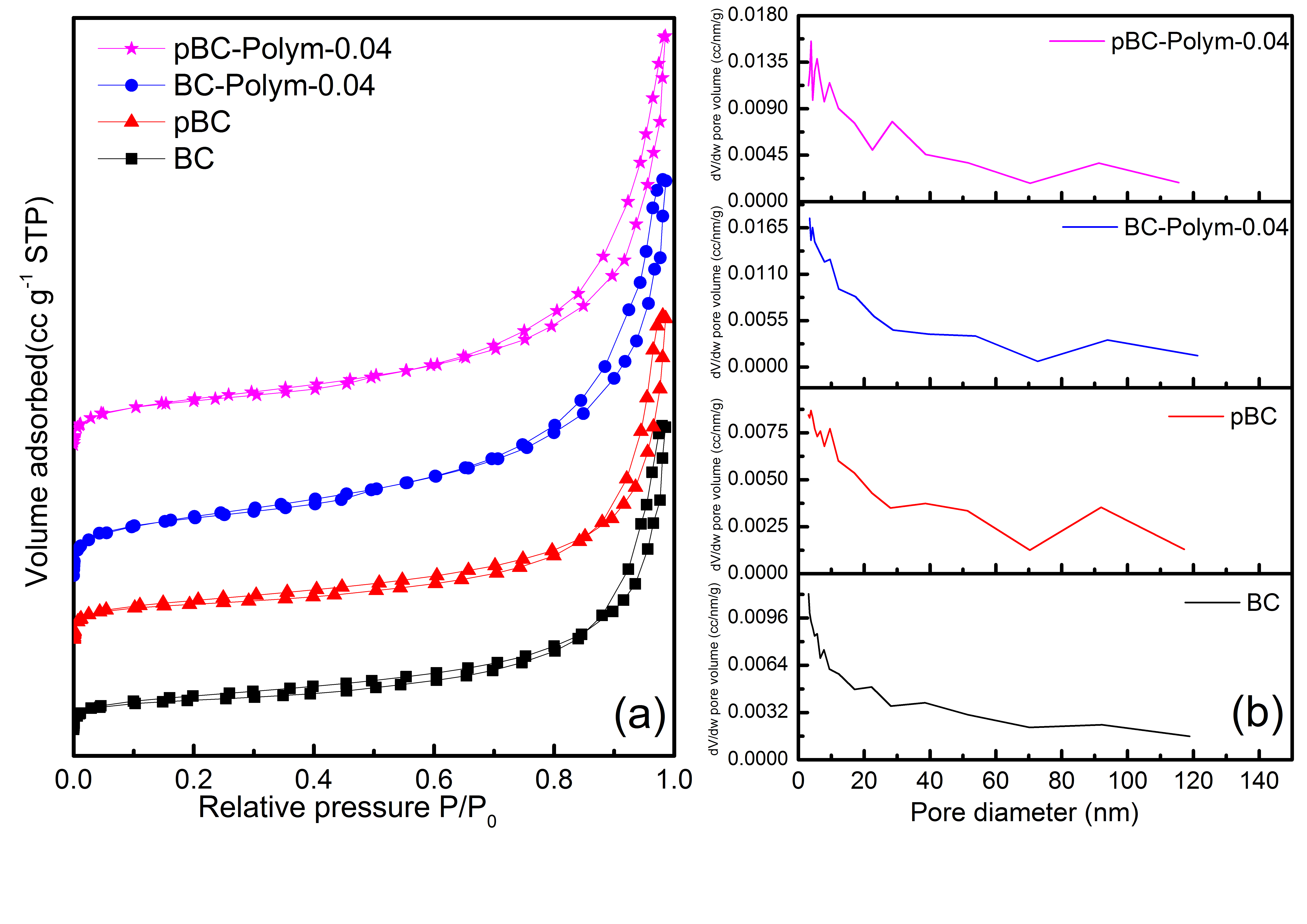


**Figure S4**. (**a**)Adsorption-desorption isotherm of BC, pBC, BC-Polym-0.04 and pBC-Polym-0.04, (**b**) pore size distribution of BC, pBC, BC-Polym-0.04 and pBC-Polym-0.04.

The porous properties were proficiently quantified by adsorption-desorption of N_2_ measurement. The isotherms and pores-size distribution for BC, pBC, BC-Polym-0.04 and pBC-Polym-0.04 were illustrated in Figure S4. It was found that the similar features of N_2_ adsorption for all four samples, combining with the type II characteristic (Figure S4a). At relative pressure of lower than 0.05, a violent rise was attributed to the abundant micropores of samples. The shorter hysteresis loop of pBC compared with BC inferred the presence of mesopores [1]. Nonetheless, the slight decline of surface area for p-BC was implicated in the consumption of micropores induced by formation of SiO_2_. The higher N_2_ adsorption of BC-Polym-0.04 and pBC-Polym-0.04 was mainly ascribed to the increase of exposure surface related to the nanostructure of poly Schiff base. The pores-size distribution presented in Figure S4b further validate the above result. In comparison with BC, the macroporous structure was presented and the pore diameter was obviously increased after modification by SiO_2_ with the close pore volume, attributed to the localized breakage and stretch of BC fibers. For BC-Polym-0.04 and pBC-Polym-0.04 composites, the deposition of poly Schiff base entirely decreased the pore size and increased the pore volume. It was ascribed to the filling the mesopores and micropores by polymers. The discrimination between BC-Polym-0.04 and pBC-Polym-0.04 was the better preservation of mesoporous structure for pBC-Polym-0.04, owing to its inherent porosity.

5. Thermal stability


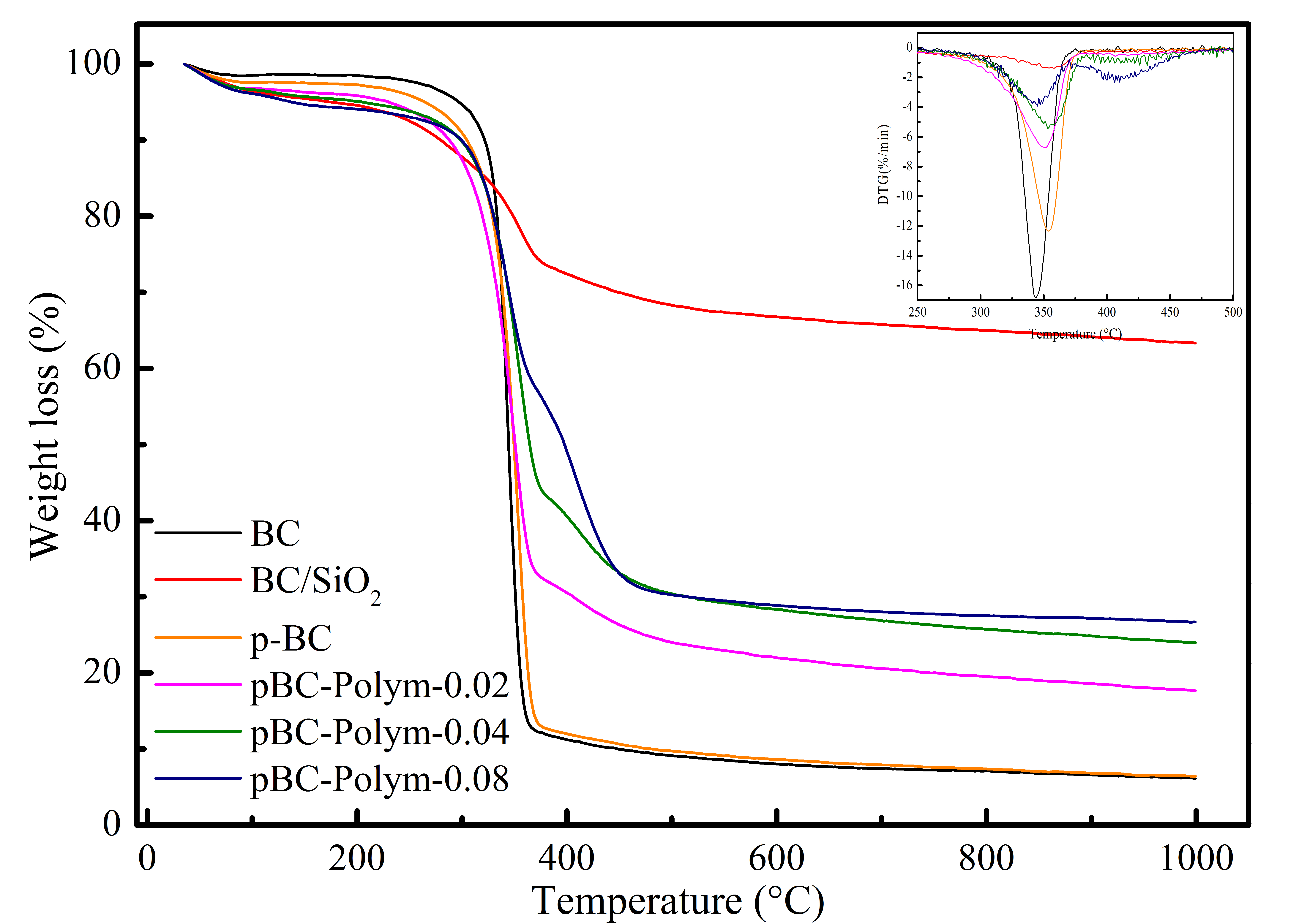


**Figure S5.tif** TGA curve of BC, BC/SiO_2_, pBC, pBC-Polym-0.02, pBC-Polym-0.04 and pBC-Polym-0.08, right inset: the DTG curves

Thermal stability of raw BC, pBC and their composites was assessed by thermogravimetric (TG) curves shown in Figure S5. The tiny weight loss for all samples before 100 ºC was caused by moisture evaporation. For raw BC, a drastic descend at 290~360 ºC with residual mass of 6.12% was attributed to the decomposition of cellulose backbone. The thermal stability of BC/SiO_2_ composites were distinctly enhanced, presenting the gradual pyrolysis process with raised temperature and 63.3% residual, involving the interaction between SiO_2_ and hydroxyl group. The incorporation of SiO_2_, however, could loosen the closely packed fibrils and reduce the amount of effective hydrogen bond by insertion of fiber bundles, resulting the onset thermal degradation of partial cellulose at lower temperature [2]. The almost same TG curve of pBC as BC suggested that the SiO_2_ served as the porogen without alteration of the molecular structure of BC, consistence of the analysis of FTIR spectrum. Similar to BC/SiO_2_, the earlier onset degradation of pBC-Polym-0.08 composites was ascribed to the weaken of hydrogen bond in cellulose molecular chain(9). Specially, two degradation stages were displayed at 220~440 ºC. The second stage of mass loss, occurring at 380~440 ºC, was related the decomposition of deposited poly Schiff base. Owing to the high thermal stability of benzenoid structure, the residual weight of composites was raised with the increased mass fraction of poly Schiff base. Still, the relative high residual was not able to be explained as the respective residual weight of poly Schiff base and BC region. It was assumed that the thermal stability of composites was enhanced by the hydrogen bond between the amine group on polymers and hydroxyl on BC. In addition, the BC fibers were assumedly insulated by the encapsulation of polymer. For the pBC-Polym-0.08, the close residual to pBC-Polym-0.08was probably induced by the superfluously stacked polymer without the consolidation by hydrogen bond.

6. Effect of pH


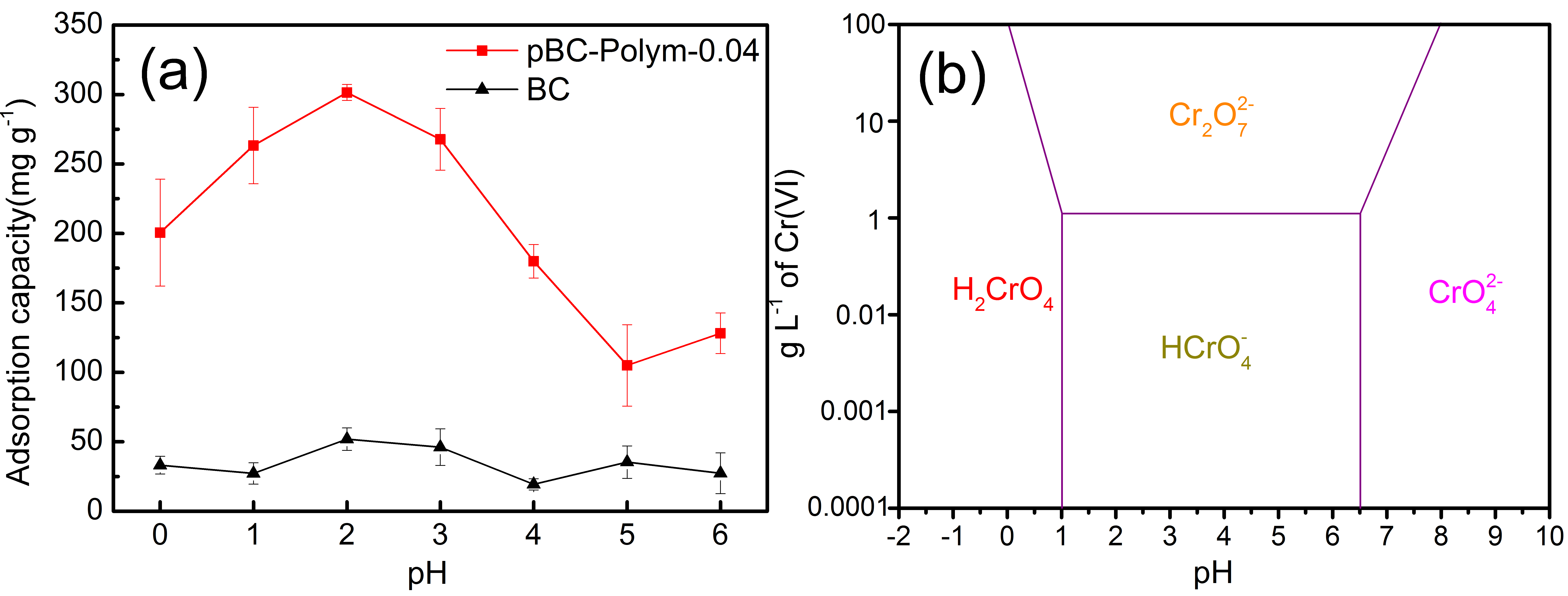


**Figure S6.tif** (**a**) Effect of pH on Cr(VI) adsorption. (**b**)Relative distribution of Cr(VI) species in water as a function of pH and Cr(VI) concentration.

According to the previous study, the speciation of Cr(VI) in aqueous can be categorized into: (1) dihydrochromate (H_2_CrO_4_) and dichromic acid(H_2_Cr_2_O_7_), dominated in the solution at pH< 2; (2) dichromate (Cr_2_O_7_^2 ̶^ ) and hydrochromate (HCrO_4_ ^̶^ ), dominant in solution at pH 2~6 and (3) chromate (CrO_4_^2 ̶^ ), dominant in alkaline solution at pH>6. With the extremely high concentration of H^+^, the electrically neutral H_2_CrO_4_ and H_2_Cr_2_O_7_ repressed the electrostatic attraction of Cr(VI) to surface of adsorbent [3-5].

The effect of pH on Cr(VI) adsorption was conducted in the pH range of 0~6. As seen in Figure S6a, the adsorption capacity reaches maximum when pH is 2. This can be explained by the speciation of Cr(VI) at different pH, which was summarized in Figure S6b. At pH 2, the protonated -N= and -NH- trapped negatively charged HCrO_4_ ^̶^ and Cr_2_O_7_^2 ̶^  via electrostatic attraction. However, when the pH is too low or too high, the ionization of Cr(VI) or the protonation degree of polymer would be suppressed, respectively, which in turn decline the performance.

7. Adsorption mechanism


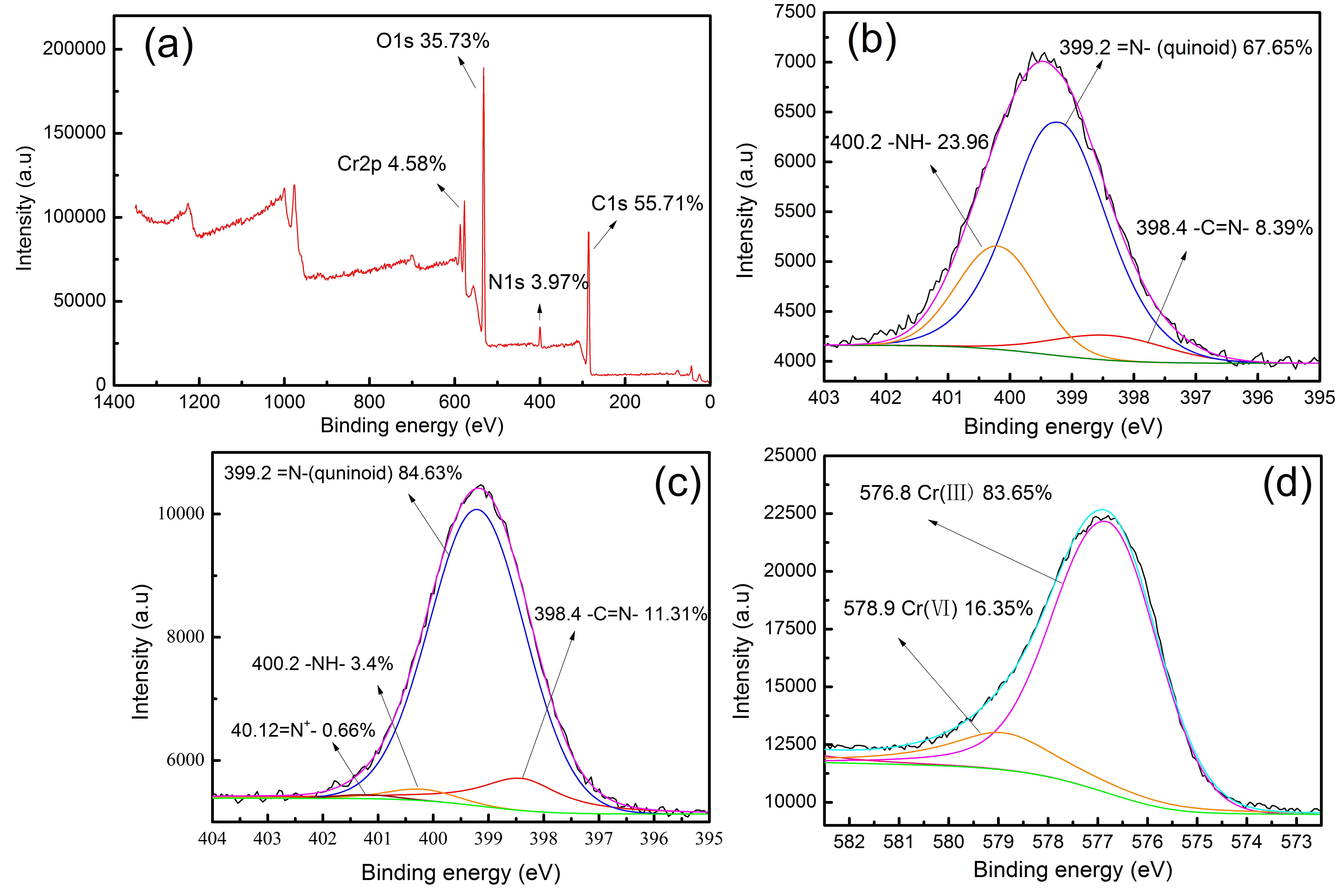


**Figure S7.** (**a)** XPS spectrum of pBC-Polym-0.04 after adsorption of Cr(VI). XPS N1s spectra of pBC-Polym-0.04 before (**b**) and after Cr(VI) adsorption (**c**); (**d)** XPS Cr2p3/2 spectra of pBC-Polym-0.04 after Cr(VI) adsorption.

On basis of former study, it was conjectured that the adsorption was initiated by the electrostatic attraction between the positively charged -N^+^=/-NH_2_^+^- and negatively-charged Cr(VI). The mechanism of Cr(VI) adsorption on pBC-Polym composite was further investigated by analysis of the pBC-Polym-0.04 before and after adsorption on a XPS. The spectrum depicted in Figure S7a shows that the peak of Cr(VI) was apparently observed after adsorption, suggesting the binding of Cr(VI) with adsorbent. Further survey for the N1s was shown in Figure S7b, c and Cr2p was displayed in Figure S7d. It can be seen that before adsorption, the N1s was divided into three states: C=N at 398.4 eV, C=N (quinoid imine) at 399.2 eV, and -NH- at 400.2 eV, with the molar ratio of 8.39%, 67.65% and 23.96%, respectively. Through the reduction of poly Schiff base, most of C=N structure was converted into C-NH- . Owing to its strong reducibility, partial benzenoid amine was oxidized to the quinoid imine structure. After adsorption, the molar ratio of C=N (quinoid imine) increased to 84.63%, -NH- diminished from 3.4%. Notably, after adsorption a new peak ascribed to the protonated quinoid imine appeared at 401.2 eV, validating the doping of proton on imine group. Additionally, the valence state of Cr on adsorbent was also investigated by XPS after Cr(VI) adsorption. The deconvolution of Cr2p peak was assigned to the peak at 578.6 eV for Cr(VI) and the peak at 576.6 eV for Cr(III) . The molar ratios of Cr(VI) and Cr(III) were 15.96% and 83.65%, respectively. From the decrement of C=N in contrast to the increment of quinoid imine as well as transition of Cr(VI) to Cr(III), the redox reaction in the adsorption was concluded. The process is depicted in Scheme 1 and could be illustrated as follows: Firstly, the Cr(VI) was electrostatic attracted onto the surface of polymer by the protonated -N^+^=/-NH_2_^+^-. Thereafter, the benzenoid amine was oxidized to the quinoid imine structure by Cr(VI). Finally, Cr(III), reduction product of Cr(VI), was chelated on polymer molecular chain by quinoid imine. The mechanism was further validated by the absence of Cr(III) in the solution after adsorption. It should be noted that the stability of poly Schiff base was strengthened by the interaction with BC molecular chain, preventing its degradation in the solution.

8. Other pollutants





**Figure S8.** removal efficiency of dynamic adsorption and maximum adsorption capacity calculated from Langmuir model of pBC-Polym-0.04 for Cu(II), Re(VII), Conga red and Orange G (**d**); adsorption isotherm of Cu(II), Re(VII), Conga red and Orange G on pBC-Polym-0.04 (**e**).

The versatility of this adsorbent toward various typical pollutants (e.g., Cu(II), Re(VII), Conga red and Orange G) was investigated by both dynamic adsorption and batch adsorption. The removal rate for each pollutant was shown in Figure 6d. Based on the isotherm experiments and calculation of Langmuir model (Figure 6e), the maximum adsorption capacity of pBC-Polym-0.04 reaches 256.4, 153.8, 333.3 and 370.3 mg g-1 toward Cu(II), Re(VII), Conga red and Orange G, respectively.

Reference

1. Li, Guiyang, Biao Zhang, Jun Yan, and Zhonggang Wang. "Micro- and Mesoporous Poly(Schiff-Base)S Constructed from Different Building Blocks and Their Adsorption Behaviors Towards Organic Vapors and Co2gas." *J. Mater. Chem. A* 2, no. 44 (2014): 18881-88.

2. Sai, H., R. Fu, L. Xing, J. Xiang, Z. Li, F. Li, and T. Zhang. "Surface Modification of Bacterial Cellulose Aerogels' Web-Like Skeleton for Oil/Water Separation." *ACS Appl Mater Interfaces* 7, no. 13 (2015): 7373-81.

3. Dinker, Manish Kumar, and Prashant Shripad Kulkarni. "Recent Advances in Silica-Based Materials for the Removal of Hexavalent Chromium: A Review." *Journal of Chemical & Engineering Data* 60, no. 9 (2015): 2521-40.

4. Mohan, D., and C. U. Pittman, Jr. "Activated Carbons and Low Cost Adsorbents for Remediation of Tri- and Hexavalent Chromium from Water." *J Hazard Mater* 137, no. 2 (2006): 762-811.

5. Chen, Jian Hua, Hai Tao Xing, Hong Xu Guo, Wen Weng, Shi Rong Hu, Shun Xing Li, Yi Hong Huang, Xue Sun, and Zhen Bo Su. "Investigation on the Adsorption Properties of Cr(Vi) Ions on a Novel Graphene Oxide (Go) Based Composite Adsorbent." *J. Mater. Chem. A* 2, no. 31 (2014): 12561-70.
